# Supplementary material for: Behavioural analysis of factors influencing prescribing for neurodegenerative diseases: A rapid review
Source: PLoS One. 2025 May 6;20(5):e0322324. doi: 10.1371/journal.pone.0322324 (PMC12054879; doi:10.1371/journal.pone.0322324)
Supplement: S1 Appendix — (DOCX) [file pone.0322324.s001.docx]

## S1 Appendix. Full inclusion and exclusion criteria.

| Inclusion criteria | Exclusion criteria |
| --- | --- |
| - All papers must be related to a ND/ Parkinson’s disease and link to the following:  1. Prescribing trends only if factors/behaviours are reported to influence prescribing outcome 2. Comorbidities if they are linked to a primary ND and prescribing outcome 3. Labels/advertising that influence decision to prescribe 4. Interventions, if the intervention changes prescribing outcome (e.g., educational interventions)  - Behaviours/factors influencing HCP prescribing decisions, such as:  1. Disease indication or off label use 2. Patient behaviours (e.g., drug preference) 3. Patient characteristics 4. Psychological factors (e.g., bias, affect, habit) 5. Cultural factors (e.g., peer influence, patient pressure for HCP to prescribe, joint decision making) 6. Environmental factors – policies or availability of the drug 7. Adherence – only when it is indicated to influence HCP decisions to prescribe | - Papers unrelated to a ND/Parkinson’s disease - Case studies - Book chapters - Commentaries - Articles not in English language - Studies that do not have an abstract - Animal studies - Drug trials - Non-drug prescriptions (e.g., exercise therapy/imagining tests) - Studies on unlicenced or experimental drugs - Pharmacological studies (e.g., that describe the efficacy/safety of a drug and do not indicate factors that influence prescribing) - Prevalence papers that only describe increase or decrease of prescribing with no clear linked factors - Adherence papers – unless there is an explicit link to influencing prescribing decisions - Drug cost analyses - Interventions with outcomes unrelated to changing prescribing decisions - Literature reviews |

HCP, healthcare professional; ND, neurodegenerative disease.
