# Supplementary material for: Behavioural analysis of factors influencing prescribing for neurodegenerative diseases: A rapid review
Source: PLoS One. 2025 May 6;20(5):e0322324. doi: 10.1371/journal.pone.0322324 (PMC12054879; doi:10.1371/journal.pone.0322324)
Supplement: S2 Appendix — (DOCX) [file pone.0322324.s002.docx]

## S2 Appendix. Full search strategy for Ovid MEDLINE.

**Ovid MEDLINE** (search performed on 04/03/2024)

1. Exp health personnel/
2. Health* adj3 (personnel OR professional* OR worker* OR provider*)
3. clinician* or consultant* or doctor* or family practition* or general practition* or gp or nurse* or pharmacist* or physician* or neurologist*
4. 1 or 2 or 3
5. exp Drug Prescriptions/ OR exp Pharmacy Administration/
6. prescri* adj4 (attitude* or variation* or behavio$r or pattern* or practice* or habit* or chang* or rational* or influenc* OR decision* OR decide* OR barrier* OR belief* OR choice*)
7. (drug* adj2 dispens*)
8. 5 or 6 or 7
9. exp Parkinsonian Disorders/
10. Parkinson* OR antiparkinson
11. 9 or 10
12. 4 and 8 and 11
13. Exp Neurodegenerative Diseases/
14. (Subacute Spongiform Encephalopath*) OR ((Creutzfeldt OR Jakob) AND (disease* OR syndrome*)) OR (Huntington*) OR (Chronic Progressive Hereditary Chorea) OR (Motor Neuron Disease*) OR (Motor System Disease*) OR (Lateral Scleros*) OR (Anterior Horn Cell Disease) OR (multiple sclerosis) OR (Disseminated Sclerosis) OR (Pick* Disease) OR (Lobar Atrophy Brain) OR (Binswanger* Disease) OR (Chronic Progressive Subcortical Encephalopathy) OR (Binswanger* Encephalopathy) OR (Subcortical Leukoencephalopath*) OR (Encephalopathy Subcortical Arteriosclerotic) OR (Neurodegenerative Disease*) OR (Neurologic Degenerative Disease*) OR (Nervous System Degenerative Diseases) OR (Neurodegenerative Disorder*) OR (Neurologic Degenerative Condition*) OR (Degenerative Neurologic Disorder*) OR (Degenerative Diseases, Spinal Cord) OR Parkinson* OR (Paralysis Agitans) OR Alzheimer* OR Dementia
15. 13 or 14
16. 4 and 8 and 15

Limit 16 to (English language and humans)
