# Supplementary material for: Behavioural analysis of factors influencing prescribing for neurodegenerative diseases: A rapid review
Source: PLoS One. 2025 May 6;20(5):e0322324. doi: 10.1371/journal.pone.0322324 (PMC12054879; doi:10.1371/journal.pone.0322324)
Supplement: S3 Appendix — (DOCX) [file pone.0322324.s003.docx]

## S3 Appendix. TDF codebook.

| **TDF domains (Cane et al., (2012)** | **Original definitions** | **Coding rules** | **Coding examples** |
| --- | --- | --- | --- |
| Knowledge | An awareness of the existence of something | Reference to already having knowledge, lack of knowledge or resource of knowledge (e.g., guidelines/scientific literature) | *Healthcare workers and family members were aware that antipsychotics cause side effects. Non-consultants acknowledge their own limited knowledge on this topic and welcome further education* (Walsh et al., 2018) |
| Skills | An ability or proficiency acquired through practice | Reference to having or attending specific training courses or experience of practice | *Compounding the complexity surrounding prescribing decisions… was the fact that health professionals felt they rarely received adequate training in how to 'stop' medications* (Cross et al., 2020) |
| Social/ professional role and identity | A coherent set of behaviours and displayed personal qualities of an individual in a social or work setting | Self-perceived level of specialism or defined professional role | *Enabling characteristics describe the ability and access to secure health care services and include physician speciality (neurologist V non-neurologists)* (Earla et al., 2020) |
| Beliefs about capabilities | Acceptance of the truth, or validity about an ability, talent or facility that a person can put to constructive use | Recognised competency or self-efficacy to preform/manage certain tasks. | *A desire to exercise clinical experience and judgement was a recurrent theme* (Duthie 2011) |
| Optimism | The confidence that things will happen for the best or that desired goals will be attained | Expectation of positive outcomes | No applicable data found |
| Beliefs about consequences | Acceptance of the truth, or validity about outcomes of a behaviour in a given situation | Reported beliefs of potential negative outcomes, the likelihood of increased risk or fallacious beliefs | *The aim of this study was to explore the use of fallacious arguments in professionals' deliberations about antipsychotic prescribing* (Donyai et al., 2017) |
| Reinforcement | Increasing the probability of a response by arranging a dependent relationship or contingency between the response and a given stimulus | Discussion around having a positive impact that encouraged prescribing behaviour | No applicable data found |
| Intentions | A conscious decision to perform a behaviour or a resolve to act in a certain way | Assertive decision or plan to act | No applicable data found |
| Goals | Mental representations of outcomes or end states that an individual wants to achieve | Discussion of a desirable aim or achievable outcomes | No applicable data found |
| Memory, attention and decision making | The ability to retain information, focus selectively on aspects of the environment and choose between two or more alternatives | Biopsychosocial patient factors that inform decision making/action, recalled physician knowledge, concentration/attention to tasks | *Factors that impact on this decision include certainty of diagnosis, balance of prognostic factors and patient preference* (Broadley et al., 2014)  *Professionals balanced clinical factors including individual disease characteristics and non-clinical factors including patient preference for each type of decision* (van den Heuvel et al., 2022a) |
| Environmental context and resources | Any circumstance of a person’s situation or environment that discourages or encourages the development of skills and abilities, independence, social competence and adaptive behaviour | Discussion around convenience of administrating medication, cost, assess, environmental setting or geographical residency | *Safety and costs are also crucial to consider, other factors like individual experience, marketing pressure, socio-economical environment, patients needs and expectations have their own influence* (Rascol et al., 2002) |
| Social influence | Those interpersonal processes that can cause individuals to change their thoughts, feelings or behaviours | Peer, carer, patient preference or opinion that influences a decision, additionally, changing guidelines, media exposure and discussion around prescribing cultures | *Prescribing practices are influenced by organisational prescribing cultures, informal benchmarking within peer networks… prescribers in England felt most constrained by guidelines* (Cameron et al., 2019) |
| Emotion | A complex reaction pattern, involving experiential, behavioural, and physiological elements, by which the individual attempts to deal with a personally significant matter or event | Reports of an evoked emotional reaction such as anxiety, fear, stress, distress that influence prescribing | *Staff distress at patients’ agitation was associated with antipsychotic and anxiolytic drug use* (Zuidema et al., 2011) |
| Behavioural regulation | Anything aimed at managing or changing objectively observed or measured actions. | Discussion of check lists or prompts served as a reminder to change prescribing | No applicable data found |

TDF, Theoretical Domains Framework.
