# Supplementary material for: Behavioural analysis of factors influencing prescribing for neurodegenerative diseases: A rapid review
Source: PLoS One. 2025 May 6;20(5):e0322324. doi: 10.1371/journal.pone.0322324 (PMC12054879; doi:10.1371/journal.pone.0322324)
Supplement: S4 Appendix — (DOCX) [file pone.0322324.s004.docx]

## S4 Appendix. Quality assessment using the Mixed Methods Appraisal tool.

| Studies | Criteria from the Mixed Methods Appraisal Tool | | | | | | | | | | | | | | | | | | | |
| --- | --- | --- | --- | --- | --- | --- | --- | --- | --- | --- | --- | --- | --- | --- | --- | --- | --- | --- | --- | --- |
|  | 1.1 | 1.2 | 1.3 | 1.4 | 1.5 | 3.1 | 3.2 | 3.3 | 3.4 | 3.5 | 4.1 | 4.2 | 4.3 | 4.4 | 4.5 | 5.1 | 5.2 | 5.3 | 5.4 | 5.5 |
| Qualitative studies |  | | | | | | | | | | | | | | | | | | | |
| Cameron et al., (2019) | 2 | 2 | 2 | 2 | 2 |  |  |  |  |  |  |  |  |  |  |  |  |  |  |  |
| Cross et al., (2020) | 2 | 2 | 2 | 2 | 2 |  |  |  |  |  |  |  |  |  |  |  |  |  |  |  |
| Disalvo et al., (2020) | 2 | 2 | 2 | 2 | 2 |  |  |  |  |  |  |  |  |  |  |  |  |  |  |  |
| Donyai et al., (2017) | 2 | 2 | 2 | 2 | 2 |  |  |  |  |  |  |  |  |  |  |  |  |  |  |  |
| Gill et al., (2019) | 2 | 2 | 2 | 2 | 2 |  |  |  |  |  |  |  |  |  |  |  |  |  |  |  |
| Green et al., (2019) | 2 | 2 | 2 | 2 | 2 |  |  |  |  |  |  |  |  |  |  |  |  |  |  |  |
| Kerns et al., (2018) | 2 | 2 | 2 | 2 | 2 |  |  |  |  |  |  |  |  |  |  |  |  |  |  |  |
| Smeets et al., (2014) | 2 | 2 | 2 | 2 | 2 |  |  |  |  |  |  |  |  |  |  |  |  |  |  |  |
| van den Heuvel et al., (2022a) | 2 | 2 | 2 | 2 | 2 |  |  |  |  |  |  |  |  |  |  |  |  |  |  |  |
| Walsh et al., (2018) | 2 | 2 | 2 | 2 | 2 |  |  |  |  |  |  |  |  |  |  |  |  |  |  |  |
| Wood-Mitchell et al., (2008) | 0 | 2 | 2 | 2 | 2 |  |  |  |  |  |  |  |  |  |  |  |  |  |  |  |
| Nijhuis et al., (2016) | 2 | 2 | 2 | 2 | 2 |  |  |  |  |  |  |  |  |  |  |  |  |  |  |  |
| Quantitative non-randomised studies |  | | | | | | | | | | | | | | | | | | | |
| Bell et al., (2020) |  |  |  |  |  | 2 | 2 | 2 | 0 | 2 |  |  |  |  |  |  |  |  |  |  |
| Degli et al., (2016) |  |  |  |  |  | 2 | 2 | 2 | 2 | 2 |  |  |  |  |  |  |  |  |  |  |
| Desai et al., (2019) |  |  |  |  |  | 2 | 2 | 2 | 2 | 2 |  |  |  |  |  |  |  |  |  |  |
| Earla et al., (2020) |  |  |  |  |  | 2 | 2 | 2 | 2 | 2 |  |  |  |  |  |  |  |  |  |  |
| Gardette et al., (2014) |  |  |  |  |  | 2 | 2 | 0 | 2 | 2 |  |  |  |  |  |  |  |  |  |  |
| Hillmer et al., (2006) |  |  |  |  |  | 2 | 2 | 2 | 2 | 1 |  |  |  |  |  |  |  |  |  |  |
| Hoffmann et al., (2011) |  |  |  |  |  | 2 | 2 | 2 | 2 | 2 |  |  |  |  |  |  |  |  |  |  |
| Jani et al., (2001) |  |  |  |  |  | 2 | 2 | 0 | 0 | 1 |  |  |  |  |  |  |  |  |  |  |
| Jeschke et al., (2011) |  |  |  |  |  | 2 | 2 | 2 | 2 | 2 |  |  |  |  |  |  |  |  |  |  |
| Martin et al., (1999) |  |  |  |  |  | 2 | 2 | 0 | 0 | 2 |  |  |  |  |  |  |  |  |  |  |
| Martinez-Lage et al., (2010) |  |  |  |  |  | 2 | 2 | 1 | 1 | 2 |  |  |  |  |  |  |  |  |  |  |
| McIlroy et al., (2015) |  |  |  |  |  | 2 | 2 | 2 | 2 | 2 |  |  |  |  |  |  |  |  |  |  |
| Monette et al., (2013) |  |  |  |  |  | 2 | 2 | 2 | 2 | 2 |  |  |  |  |  |  |  |  |  |  |
| Neo et al., (2020) |  |  |  |  |  | 2 | 2 | 2 | 2 | 2 |  |  |  |  |  |  |  |  |  |  |
| Orayj et al., (2021) |  |  |  |  |  | 2 | 2 | 2 | 1 | 2 |  |  |  |  |  |  |  |  |  |  |
| Ott et al., (2023) |  |  |  |  |  | 2 | 2 | 2 | 2 | 2 |  |  |  |  |  |  |  |  |  |  |
| Podhorna et al., (2020) |  |  |  |  |  | 2 | 2 | 2 | 0 | 2 |  |  |  |  |  |  |  |  |  |  |
| Rochon et al., (2018) |  |  |  |  |  | 2 | 2 | 2 | 2 | 2 |  |  |  |  |  |  |  |  |  |  |
| Thomas et al., (2013) |  |  |  |  |  | 2 | 2 | 2 | 1 | 2 |  |  |  |  |  |  |  |  |  |  |
| Crispo et al., (2015) |  |  |  |  |  | 2 | 2 | 2 | 2 | 2 |  |  |  |  |  |  |  |  |  |  |
| Schroder et al., (2011) |  |  |  |  |  | 2 | 2 | 2 | 1 | 2 |  |  |  |  |  |  |  |  |  |  |
| Ooba et al., (2011) |  |  |  |  |  | 2 | 2 | 2 | 1 | 1 |  |  |  |  |  |  |  |  |  |  |
| Trifiro et al., (2008) |  |  |  |  |  | 2 | 2 | 2 | 1 | 2 |  |  |  |  |  |  |  |  |  |  |
| Tan et al., (2005) |  |  |  |  |  | 2 | 2 | 2 | 0 | 2 |  |  |  |  |  |  |  |  |  |  |
| Grandas and Kulisevsky (2003) |  |  |  |  |  | 2 | 1 | 2 | 0 | 2 |  |  |  |  |  |  |  |  |  |  |
| Zuidema et al., (2011) |  |  |  |  |  | 2 | 2 | 2 | 2 | 2 |  |  |  |  |  |  |  |  |  |  |
| Wei et al., (2015) |  |  |  |  |  | 2 | 2 | 2 | 2 | 2 |  |  |  |  |  |  |  |  |  |  |
| Houghton et al., (2019) |  |  |  |  |  | 2 | 2 | 2 | 2 | 2 |  |  |  |  |  |  |  |  |  |  |
| Esposti et al., (2017) |  |  |  |  |  | 2 | 2 | 2 | 0 | 2 |  |  |  |  |  |  |  |  |  |  |
| Quantitative descriptive |  | | | | | | | | | | | | | | | | | | | |
| Cousins et al., (2017) |  |  |  |  |  |  |  |  |  |  | 2 | 2 | 2 | 2 | 2 |  |  |  |  |  |
| Dhuny et al., (2020) |  |  |  |  |  |  |  |  |  |  | 2 | 2 | 2 | 2 | 2 |  |  |  |  |  |
| Fargel et al., (2007) |  |  |  |  |  |  |  |  |  |  | 2 | 2 | 2 | 1 | 2 |  |  |  |  |  |
| Hanson et al., (2014) |  |  |  |  |  |  |  |  |  |  | 1 | 2 | 2 | 1 | 2 |  |  |  |  |  |
| Oremus et al., (2007) |  |  |  |  |  |  |  |  |  |  | 2 | 2 | 2 | 2 | 2 |  |  |  |  |  |
| Stephens et al., (2014) |  |  |  |  |  |  |  |  |  |  | 2 | 2 | 2 | 0 | 2 |  |  |  |  |  |
| van den Heuvel et al., (2022b) |  |  |  |  |  |  |  |  |  |  | 2 | 2 | 2 | 2 | 2 |  |  |  |  |  |
| Walker et al., (2018) |  |  |  |  |  |  |  |  |  |  | 2 | 2 | 2 | 2 | 2 |  |  |  |  |  |
| Werner (2006) |  |  |  |  |  |  |  |  |  |  | 2 | 2 | 2 | 1 | 2 |  |  |  |  |  |
| Mixed method studies |  | | | | | | | | | | | | | | | | | | | |
| Duthie et al., (2011) |  |  |  |  |  |  |  |  |  |  |  |  |  |  |  | 0 | 0 | 0 | 0 | 0 |
| Peisah et al., (2015) |  |  |  |  |  |  |  |  |  |  |  |  |  |  |  | 2 | 2 | 2 | 2 | 2 |
| Petrazzuoli et al., (2020) |  |  |  |  |  |  |  |  |  |  |  |  |  |  |  | 2 | 2 | 2 | 2 | 2 |
| Timotijevic et al., (2020) |  |  |  |  |  |  |  |  |  |  |  |  |  |  |  | 2 | 2 | 2 | 0 | 2 |
|  |  |  |  |  |  |  |  |  |  |  |  |  |  |  |  |  |  |  |  |  |

Key: 0 = no evidence available, 1 = cannot tell if there is evidence, 2 = evidence available
