# Supplementary material for: Behavioural analysis of factors influencing prescribing for neurodegenerative diseases: A rapid review
Source: PLoS One. 2025 May 6;20(5):e0322324. doi: 10.1371/journal.pone.0322324 (PMC12054879; doi:10.1371/journal.pone.0322324)
Supplement: S5 File — (DOCX) [file pone.0322324.s005.docx]

## Statement on industrial partnership

Alpharmaxim, a long-established specialised healthcare marketing agency, identified a unique opportunity to revolutionise healthcare communications campaigns, using innovative application of behavioural science research. This is important because pharmaceutical company’s marketing campaigns for drugs have followed the same model for years (using the same templates out of habit) and there are currently limited means to measure their success. Alpharmaxim is passionate about driving change in how healthcare communications campaigns are developed, by focusing on identifying beliefs and barriers that lead to behavioural changes, enabling much-needed drugs to reach patients.

Alpharmaxim already has strong differentiation from its competitors with its focus on rare diseases and an established history of harnessing behavioural psychology. Alpharmaxim’s unique Belief Continuum ^©^ model uses methodology derived from the work of Nobel Prize-winning psychologist Professor Daniel Kahneman to nudge healthcare professionals to progress from an underlying inhibitory belief to one that allows them to embrace the true potential of a drug.

Alpharmaxim is building a cutting-edge tool to bolster its position as a leader in behavioural change: the Healthcare Belief/Barrier Identification Tool (H-BIT). This innovative tool emerges from an in-depth research initiative designed to assess how behavioural science principles can influence prescription decisions. The current report lays the groundwork for a robust, multi-faceted approach that integrates rapid systematic reviews, focus groups, and psychometric analyses, to better understand and enhance the decision-making processes driving healthcare professionals’ prescription behaviours.

H-BIT’s inaugural application focuses on Parkinson’s disease, a strategic choice given the condition’s prevalence and the established reliance on levodopa therapy. These factors provide a rich and well-defined framework for exploring prescription decision-making. The insights gained from this research not only enhance understanding of prescribing behaviours in Parkinson’s disease, but also set the stage for broader applications across other neurodegenerative disorders. This pioneering application of behavioural science to prescription practices represents a significant leap forward in current orthodoxy, demonstrating Alpharmaxim’s commitment to delivering innovative, evidence-based solutions in the healthcare domain.

Alpharmaxim supports biopharma to change some aspects of disease management and answer unmet medical needs by developing effective communications campaigns. The sector is very dependent on data and evidence. Unfortunately, the launch of a new drug or a change in therapeutic management often does not match expectations; uptake is not as predicted where logic is the sole basis of prescriber choice and frequently there are deep-seated emotional beliefs and entrenched habitual behaviours that prevent an action to change or adoption of a new behaviour.

This is a completely novel approach that has not been used in the study of healthcare professional behaviour. The subsequent publication will ensure that Alpharmaxim is fully differentiated from its competitors as a behaviour change expert by embedding the results in its core business offering.

The outcome of the H-BIT is a guide on the common barriers in a specific disease area that are likely to require change to successfully launch a drug for that disease. The overall aim is to completely disrupt the healthcare communications industry by undermining the use of habitual tactics, making the focus on behavioural change a necessity. There are potentially hundreds of different complex or difficult-to-manage diseases where the need to change behaviour is paramount, and this report is the result of the fruitful collaboration that resulted in the foundational stage of the H-BIT generation.

This project is part of a larger knowledge transfer initiative funded by the UK government, designed to foster collaboration between academia and industry to break down traditional barriers. Such initiatives operate under a structured framework to ensure equitable contributions from both academic and industry partners. Alpharmaxim’s involvement was instrumental in refining the research focus, particularly in identifying the specificity of the research question and maintaining the project’s alignment with its intended objectives. Their expertise and practical insights significantly enriched the study and ensured that our research remained targeted and impactful throughout its development.

© Alpharmaxim, All rights reserved
